# Protective role of intravesical BCG in COVID-19 severity

**DOI:** 10.1186/s12894-021-00823-6

**Published:** 2021-03-30

**Authors:** Héctor Gallegos, Pablo A. Rojas, Francisca Sepúlveda, Álvaro Zúñiga, Ignacio F. San Francisco

**Affiliations:** 1 Departamento de Urología, Facultad de Medicina, Pontificia Universidad Católica de Chile, Diagonal Paraguay 362, Santiago, Chile; 2 Servicio de Urología, Hospital Sótero del Río, Santiago, Chile; 3 Escuela de Pregrado, Facultad de Medicina, Pontificia Universidad Católica de Chile, Santiago, Chile

**Keywords:** COVID-19, Non-muscle invasive bladder cancer, Intravesical BCG

## Abstract

**Objectives:**

To establish the role of BCG instillations in the incidence and mortality of COVID-19.

**Patients and methods:**

NMIBC patients in instillations with BCG (induction or maintenance) during 2019/2020 were included, establishing a COVID-19 group (with a diagnosis according to the national registry) and a control group (NO-COVID). The cumulative incidence (cases/total patients) and the case fatality rate (deaths/cases) were established, and compared with the national statistics for the same age group. *T*-test was used for continuous variables and Fisher's exact test for categorical variables.

**Results:**

175 patients were included. Eleven patients presented CIS (11/175, 6.3%), 84/175 (48.0%) Ta and 68/175 (38.9%) T1. Average number of instillations = 13.25 ± 7.4. One hundred sixty-seven patients (95.4%) had complete induction. Forty-three patients (cumulative incidence 24.6%) were diagnosed with COVID-19. There is no difference between COVID-19 and NO-COVID group in age, gender or proportion of maintenance completed. COVID-19 group fatality rate = 1/43 (2.3%). Accumulated Chilean incidence 70–79 years = 6.3%. Chilean fatality rate 70–79 years = 14%.

**Conclusions:**

According to our results, patients with NMIBC submitted to instillations with BCG have a lower case-fatality rate than the national registry of patients between 70 and 79 years (2.3% vs. 14%, respectively). Intravesical BCG could decrease the mortality due to COVID-19, so instillation schemes should not be suspended in a pandemic.

## Introduction

The virus called severe acute respiratory syndrome coronavirus 2 (SARS-CoV-2), the cause of coronavirus disease 2019 (better known as COVID-19) has generated a pandemic never seen by mankind before [1]. Today, late July, at least 13 million people around the world have been diagnosed with COVID-19, and almost 5000 deaths are accounted daily due to the disease [2], exceeding 500 thousand deaths worldwide. In Chile, currently more than 300 thousand infected and 8 thousand deaths are described.

Major efforts focus on discovering a vaccine for the disease. In this context, the utility of Mycobacterium bovis Bacillus Calmette–Guérin (BCG) is proposed. BCG is a live attenuated vaccine for tuberculosis, and it has been described to provide protection against respiratory infections other than tuberculosis [3, 4], reducing its contagiousness, its severity and even mortality [5]. The mechanism by which BCG confers immunity to other infections is unknown [6], however, there is evidence that nonspecific protective effect is constituted by innate immune cells such as monocytes and natural killer cells, independent of T- and B-cell memory (called “trained inmmunity”) [7, 8].

Various observational studies have shown that countries with BCG vaccination programs present lower mortality rates from COVID-19 than countries without vaccination [9]. Italy and USA, countries without a vaccine program, have experienced higher mortality associated to COVID-19 than countries with long-standing universal BCG vaccination policies, such as South Korea and Japan [8]. Certainly, these relationships are observational and not necessarily causal.

Moreover, bladder instillations with BCG is a standard of care in patients with intermediate-high risk non-muscle invasive bladder cancer (NMIBC). BCG has shown a 32% reduction in bladder tumor recurrence risk and a 27% reduction in odds of progression [10].

The potential protective role of instillation of BCG in NMIBC against diagnosis and aggressiveness of COVID-19 has not been published or described in the current literature.

We aimed to observe whether intravesical BCG has protective role in terms of reduced infection by SARS-CoV-2, or, a less aggressive presentation of COVID-19 disease among NMIBC patients. We evaluated the incidence of COVID-19 and its severity in a cohort of patients with BCG bladder instillations.

## Patients and methods

### Patients

The study cohort included Chilean patients over 18 years, with histologic diagnose of NMIBC (CIS, Ta, T1) who underwent BCG treatment and had the diagnosis of COVID-19 after BCG instillations. This cohort of patients were treated with BCG instillations in 2019 and 2020 at Hospital Clínico de la Pontificia Universidad Católica de Chile. Patients were treated continuously with BCG bladder instillations and were receiving induction or maintenance at the time of the first case of COVID-19 was reported in Chile (March 3rd, 2020). Patients included were in induction or maintenance stages of their treatment as described by Morales et al. [11], with full dose (90 mg) or lower dose (30 mg) at least for 1-h intravesical instillation. Danish 1331 Mycobacterium bovis strain was used in every patient. We described all variables mentioned above and recurrence or BCG infection (BCGitis).

Patients with COVID-19 were diagnosed by real-time reverse transcription-PCR (RT-PCR) assays. The COVID-19 diagnosis notifications were obtained through the official Chilean national register (“Epivigila”), which performs immediate mandatory surveillance and notification of the COVID-19 diagnosis in all health centers in Chile.

We compared patients who underwent BCG treatment has COVID-19 diagnosis with patients without infection, to establish the influence of age, BCG dosis and induction or maintenance in reduce infection or less aggressiveness COVID-19.

Moreover, we compared our COVID-19 patients in BCG treatment with the overall population of the same age group (70–79 years), according to Chilean national register. National register of COVID-19 pandemic is complete and trustworthy [12].

### Follow-up

Prospective follow-up of the patients was carried out from the start of the pandemic in Chile until February 2021. When one of our patients was diagnosed with COVID-19, a clinical follow-up of the evolution of the disease was carried out and, in case they survived the disease, they were contacted by telephone to define the consequences of the disease, such as severity, symptoms, need for mechanical ventilation. Informed consent was obtained from all subjects contacted.

### Statistical analysis

Descriptive and analytic statistics were performed, expressing the numerical variables as measures of central tendencies and the categorical variables as percentages. Independent samples t-test (two tails) was used to compare continuous variables, Fisher's exact test or Chi-Square test to compare categorical variables. We define the cumulative incidence, as the ratio of the covid-19 infected cases within the cohort during the follow-up period (cases/total group), the case fatality rate was defined as deaths within the group of patients infected with covid-19 (death/COVID-19 infected patients). All data were analyzed with SPSS 22.0 software (IBM SPSS Software).

### Statements

Ethics committee approval was granted by the Local Institutional Ethics Review Board. All procedures involving human participants were performed in accordance with the 1975 Helsinki declaration and its later amendments.

## Results

A total of 175 patients were recruited in the study cohort. The main clinical-pathological and demographic characteristics of the group are described in Table 1.

**Table 1:** Clinical-pathological characteristics

Characteristics	Covid-19 patients (n = 43)	All BCG patients (n = 175)
BCG dosage, mg ± SD	39.64 ± 22.9	44.3 ± 25.5
BCG instillations, n ± SD	12.20 ± 8.1	13.25 ± 7.4
Induction completed, % (n)	86.0% (37)	95.4% (167)
Maintenance completed, % (n)	62.8% (27)	65.1% (114)
Maintenance therapy time, years ± SD	1.48 ± 0.83	1.11 ± 0.98
T stage		
Cis, % (n)	9.3% (4)	6.3% (11)
Ta, % (n)	55.8% (24)	48.0% (84)
T1, % (n)	34.9% (15)	38.9% (68)
Previous relapse, % (n)	55.8% (24)	42.9% (75)
BCG infection (“BCGitis”), % (n)	25.6% (11)	18.9% (33)

During the study period, 43 patients (cumulative incidence = 24.6%) were diagnosed with COVID-19. There was no statistically significant difference between COVID-19 and No-COVID patients in age (72.9 years vs. 73.1 years, *p* = 0.899), Gender (83.7% vs. 72.0%, *p* = 0.157), and proportion of patients with maintenance therapy completed (62.8% vs. 75.0%, *p* = 0.170, respectively) (Table 2).

**Table 2:** Univariate analysis between patients with COVID-19 and patients without infection

Characteristics	Covid group (n = 43)	No-covid group (n = 132)	*p* value
Mean age (years) ± SD	72.9 ± 10.9	73.1 ± 10.1	0.899
Male, % (n)	83.7% (36)	72.0% (95)	0.157
Full dose BCG, % (n)	16.3% (7)	22.0% (29)	0.518
Maintenance therapy, % (n)	62.8% (27)	75.0% (99)	0.170

In the group of COVID-infected patients (43 patients), during follow-up only 1 patient died from the disease (Case Fatality Rate = 2.3%), only 2 patient (4.6%) required hospitalization, and only 2 patients (4.6%) had COVID pneumonia. Most patients only presented upper respiratory symptoms, compatible with a flu.

According official statistics of Chile [12], in the same period of time, control group (age group 70–79 years) had a cumulative incidence of COVID-19 infection of 6.3% (40.629 COVID-19 confirmed cases, 643.423 people in the 70–79 age group), and a case fatality rate of 14% (5.671 deaths due to COVID-19, 40.629 confirmed cases).

## Discussion

As we showed previously, there are several studies that have compared the rates of infection and SARS-CoV-2 between different countries. Ozdemir et al. [7], in April 2020, demonstrated that mean cases per habitants is significantly lower in BCG-vaccinated countries than in BCG-non-vaccinated countries (*p* < 0.0001). Moreover, mean death is significantly lower in BCG-vaccinated countries compared to BCG-non-vaccinated countries (*p* < 0.0001). According to the evidence, BCG vaccination produces various pro- inflammatory cytokines, such as IL-1β, tumour necrosis factor and IL-6 [13], that explain the BCG vaccine’s protective effect against other pathogens. Furthermore, there is an ongoing trial to use BCG to prevent COVID-19 (BCG Vaccination to Protect Healthcare Workers Against COVID-19 (BRACE) trial, NCT04327206).

Intravesical BCG not only generates a bladder inflammatory response; it induces a systemic effect with increased levels of IgG. The antibody response increased gradually at 3 months after instillations are completed [14]. According to Desouky, this can raise the question whether it is possible that intra-vesical BCG treatment for NMIBC patients provided them with a beneficial effect [15].

This manuscript is the first study that correlates intravesical BCG with less case fatality rate in a cohort of patients with NMIBC. It is interesting to note that this group of cancer patients undergoing BCG instillations had a lower-case fatality rate than general population of the same age group, even considering that cancer patients are basally more fragile. Our initial results are promising, but these data should be taken with caution, given that they are based on a limited number of infected patients.

It is striking that the infection rate in this cohort of instilled patients was not lower than the rate of the general population in the same age group, it was even higher. This could indicate that perhaps the immunity associated with BCG could influence the severity of the disease more than preventing infection.

Recent evidence suggests that patients with cancer are at higher risk of infection for SARS-CoV-2 and may have poorer outcomes [16]. Thus, it has been proposed to defer instillations with BCG by at least 3 weeks due to the risk of increasing severity in patients with COVID-19 [17]. However, according to our data, intravesical BCG would be beneficial in case of infection, since it would decrease the illness’ severity (and mortality).

This study has some limitations. First, this is a cohort study with a reduced number of patients and a limited total number of infected (43 patients), which could not allow definitive conclusions to be made. Second, we cannot assure that the patients present an infection rate due to their intravesical BCG instillations or because the patients are in lockdown. The mean age was 73 years old, and most of these patients are in mandatory quarantine. Therefore, our best conclusions are in relation to the case fatality rate, since it begins with a point in common (confirmatory diagnosis of COVID-19).

## Conclusion

According to our results, patients with NMIBC in BCG bladder instillations had lower case fatality rate than same age overall population (2.3% vs. 14%, respectively), but had more cumulative incidence (24.6% to NMIBC and 6.3% to overall population). Intravesical BCG could protect against aggressive presentation of COVID-19. More studies are needed in order to confirm our findings.

## Data Availability

The datasets used and analysed during the current study are available from the corresponding author on reasonable request.
